# A multicenter, single‐arm, open‐label interventional study of adherence to brexpiprazole during switching from previous antipsychotic drugs in patients with schizophrenia or schizoaffective disorder

**DOI:** 10.1002/npr2.12416

**Published:** 2024-01-22

**Authors:** Kazuyuki Nakagome, Hisateru Tachimori, Shiro Endo, Ken Murakami, Takaharu Azekawa, Seiji Hongo, Kazunari Niidome, Yoshitsugu Kojima, Sakiko Yamada, Hideki Oi, Tomiki Sumiyoshi

**Affiliations:** ^1^ National Center of Neurology and Psychiatry Kodaira‐shi Tokyo Japan; ^2^ Department of Clinical Data Science, Clinical Research & Education Promotion Division National Center of Neurology and Psychiatry Kodaira‐shi Tokyo Japan; ^3^ Fujimidai Hospital Hiratsuka‐shi Kanagawa Japan; ^4^ Murakami Iin Tokyo Japan; ^5^ Shioiri Mental Clinic Yokosuka‐shi Kanagawa Japan; ^6^ Ichigaya Himorogi Clinic Tokyo Japan; ^7^ Nanko Kokorono Clinic Shirakawa‐shi Fukushima Japan; ^8^ Medical Affairs, Otsuka Pharmaceutical Co. Ltd. Tokyo Japan; ^9^ Department of Preventive Intervention for Psychiatric Disorders National Institute of Mental Health, National Center of Neurology and Psychiatry Kodaira‐shi Tokyo Japan

**Keywords:** D_2_ receptor antagonists, dopamine D_2_ receptor partial agonist, medication persistence rate, safety, Specific Levels of Functioning Scale

## Abstract

The rate of medication persistence was examined in patients with schizophrenia or schizoaffective disorder during switching from previously administered antipsychotics to brexpiprazole, a new dopamine D_2_ receptor partial agonist. A multicenter, single‐arm, open‐label 24‐week interventional study was conducted, consisting of two 12‐week consecutive periods: an initial switch (by plateau cross‐titration) with the subsequent period, followed by a second maintenance period. Prior antipsychotics were olanzapine or risperidone/paliperidone. The primary and secondary outcome measures were medication persistence rates after the first 12 weeks and changes from baseline in the Specific Levels of Functioning Scale (SLOF), Subjective Well‐being under Neuroleptic drug treatment Short form (SWNS), and Positive and Negative Syndrome Scale (PANSS) scores, respectively. In total, 79 patients were administered brexpiprazole and the medication persistence rate at 12 weeks was 78.5%, which was significantly higher than the predefined threshold of 65%. Regarding the prior medication, the persistence rate at 12 weeks was 84.6% for olanzapine and 72.5% for risperidone/paliperidone. Significant improvements from baseline were observed in the SLOF, SWNS, and PANSS scores. There were no adverse events of concern. Thus, brexpiprazole appeared to be a suitable antipsychotic on switching from olanzapine, risperidone, or paliperidone.

## Abstract 2

The medication persistent rate after switching from olanzapine or risperidone/paliperidone to brexpiprazole in patients with schizophrenia or schizoaffective patients was 78.5%, exceeding the predefined threshold of 65% using a longer tapering period than in the previous trial. In addition, significant improvements in functioning, well‐being, and symptoms were observed with no adverse events of concern. Brexpiprazole appeared to be a suitable antipsychotic to switch to.

## Graphical abstract

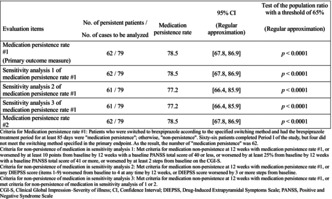

## 1 INTRODUCTION

Approximately 20 million people are reported to be affected by schizophrenia worldwide and its global burden continues to increase.^1^ Antipsychotics have been the mainstay of pharmacotherapy for schizophrenia. In order to improve symptoms and/or reduce side effects, switching of antipsychotic drugs has been investigated in schizophrenia treatment. However, it should be performed carefully to avoid the risk of worsening psychiatric symptoms associated with the switching. The method of switching should be adjusted to the type and dose of the previous and new antipsychotics, taking into consideration not only adverse events caused by the new antipsychotics but also the effects of withdrawal reactions to the previously administered antipsychotics.

Brexpiprazole has been approved for the treatment of schizophrenia and major depressive disorder in over 60 countries since its first approval in the United States in 2015.^2^ Compared with other antipsychotics, brexpiprazole requires a longer period to reach effective blood levels (10 days)^3^ and has unique pharmacological characteristics such as acting as a partial agonist at 5‐HT_1A_ and the dopamine D_2_ receptors, antagonism at 5‐HT_2A_ receptors and having low affinity for muscarinic M_1_ receptors.^4^ Brexpiprazole is a dopamine D_2_ receptor partial agonist like aripiprazole, and the strategy of switching from a previous antipsychotic to aripiprazole could be applicable in switching from a previous antipsychotic to brexpiprazole. Two points have been recommended for switching to aripiprazole from other antipsychotics.^5^, ^6^ The first point is that after aripiprazole is increased to the effective dose, it should be added to the previous drug for at least 14 days until aripiprazole has reached a steady state, followed by tapering of the previous drug. The second point is that the period of discontinuation of previous antipsychotics with strong cholinergic or histaminergic blockade should be longer than 1–2 months. There is also a tendency for brexpiprazole to cause withdrawal reactions upon rapid or sudden discontinuation of previously treated antipsychotics because of its weak binding properties to muscarinic M_1_ and histaminergic H_1_ receptors and intrinsic activity at dopamine D_2_ receptors.^4^ Therefore, it has been proposed that the occurrence of these adverse events can be reduced by tapering off the previous antipsychotic over a longer period.

Previously, a clinical trial was conducted to assess the long‐term (52‐week) safety, tolerability, and maintenance of therapeutic effect of brexpiprazole in Japanese patients with schizophrenia between 2011 and 2015.^7^ The study included patients with schizophrenia who continued treatment from a 6‐week, randomized placebo‐controlled trial, and de novo patients who switched from other antipsychotics. In the group with de novo patients, the study consisted of 2 consecutive treatment periods: a 4‐week switching period from the prior antipsychotic to brexpiprazole and a 52‐week open‐label brexpiprazole treatment period. The results showed that brexpiprazole was generally safe and well tolerated and maintained therapeutic effects in the long‐term study. However, there were still no sufficient reports on the results of switching to brexpiprazole or guidelines for switching based on experience in use. Therefore, Ishigooka et al.^8^ conducted an additional post hoc analysis of the long‐term Japanese clinical trials of brexpiprazole and examined the data of 200 patients who switched from previously treated antipsychotics. The results of the post hoc analysis showed that the discontinuation rate at the end of 8 weeks was 4.9% when aripiprazole was the prior main antipsychotic, but was 25.4% when non‐aripiprazole, dopamine D_2_ receptor antagonists were the main prior antipsychotic. This suggested that the rate of medication persistence may not be sufficient when switching from antipsychotics other than aripiprazole. However, as the switching method in the trial used a short tapering period of 2 weeks for prior antipsychotics and administration of 3–4 mg/day of brexpiprazole (above the approved dose in Japan), it did not provide sufficient reference data under actual clinical conditions in Japan. In this study, we examined the persistence rate of medication in patients with schizophrenia or schizoaffective disorder who switched from their current non‐aripiprazole antipsychotic—one of olanzapine, risperidone, or paliperidone—to brexpiprazole at 2 mg/day (the approved dose in Japan) over a period of 8 weeks. In addition, we examined the safety and efficacy of brexpiprazole after the switching.

## 2 METHODS

### 2.1 Study design and treatment

This was a multicenter, single‐arm, open‐label, interventional study conducted at 14 sites in Japan between August 1, 2018, and June 2021 (Japan Registry of Clinical Trials ID: jRCTs031180015). The work described had been carried out in the Declaration of Helsinki. All patients enrolled in the study provided written informed consent. The privacy rights of all the patients were always observed. This study consisted of two consecutive periods (total 24 weeks): period I (12 weeks; 8‐week switching period plus 4‐week post‐switch) and period II (12 weeks; weeks 12–24) (Figure S1). In period I, the prior antipsychotics were switched to brexpiprazole by first adding brexpiprazole and then, 2 weeks later, starting to reduce the dose of the prior antipsychotic. Brexpiprazole was administered orally once daily, starting at 1 mg/day, and 5–7 days later increased to 2 mg/day and continued. The dosage and administration of the previously treated antipsychotic remained unchanged until 2 weeks after the start of brexpiprazole administration, was gradually decreased over 6 weeks, and the previous antipsychotic was discontinued at 8 weeks. During period II, administration of brexpiprazole was continued at 2 mg/day.

### 2.2 Study participants

Between August 1, 2018, and December 31, 2020, a total of 87 patients were enrolled. Eligible patients were outpatients diagnosed with schizophrenia or schizoaffective disorder according to DSM‐5 and aged between 18 and 65 years as of the day of informed consent. Other inclusion criteria were as follows: patients who wished to switch the medication to a new antipsychotic in expectation of better tolerability and efficacy than that of the current antipsychotics or a reduced dosing frequency or number of concomitant medications; patients who were capable of providing written informed consent in person; patients whose chlorpromazine (CP) equivalent dose of prior antipsychotics was 150–1000 mg/day within 30 days prior to informed consent; patients whose type of prior primary antipsychotics had been unchanged within 30 days prior to informed consent; patients whose dose of prior primary antipsychotic met the following criteria on the date of informed consent: risperidone 2–8 mg, paliperidone 3–12 mg and olanzapine 5–20 mg; patients whose CP equivalent dose of all antipsychotics was 1000 mg/day or less on the day of informed consent. The antipsychotic that had a dose exceeding 50% of the total CP equivalent dose was considered to be the primary antipsychotic.

Exclusion criteria included as follows (full description of the exclusion criteria; Table S1): patients who had used clozapine; patients with physical complications of clinical concern; patients who had undergone electroconvulsive therapy within 60 days prior to informed consent; patients who had received long‐acting injectable antipsychotics within 90 days prior to informed consent; patients who had been hospitalized due to psychiatric symptoms within 90 days prior to informed consent; patients who were considered to be at a high risk of suicide.

Criteria for discontinuation (withdrawal criteria; see Table S1 for full description) included failure to increase the dose to 2 mg/day within 7 days of starting brexpiprazole, failure to discontinue previous antipsychotics after 8 weeks of brexpiprazole administration, and dose reduction to 1 mg/day after increasing the dose of brexpiprazole to 2 mg/day.

Adrenaline, CYP2D6, and CYP3A4 inhibitors were considered as concomitantly prohibited drugs, and electroconvulsive therapy and newly added psychotherapy were considered as concomitantly prohibited treatments.

Additional antipsychotics except for previous antipsychotics and brexpiprazole were considered concomitantly restricted medications, permitted at a CP equivalent dose of less than 100 mg/day for up to 3 days in each period as a treatment for adverse events.

### 2.3 Efficacy and safety assessments

The primary outcome measure was the medication persistence rate #1 at 12 weeks after starting treatment with brexpiprazole. For medication persistence rate #1, patients who were switched to brexpiprazole according to the specified switching method and received brexpiprazole for at least 85 days were considered “medication persistence.” The use of concomitantly restricted medications was considered as non‐persistence. Major secondary outcome measures were as follows: (1) change from baseline in Specific Levels of Functioning Scale (SLOF), (2) change from baseline in Subjective Well‐being under Neuroleptic drug treatment Short form (SWNS), and (3) medication persistence rate #2 at 12 weeks after initiation of treatment with brexpiprazole. For medication persistence rate #2, patients who were switched to brexpiprazole according to the specified switching method and received brexpiprazole for at least 85 days were considered medication persistence. However, unlike #1, the use of concomitantly restricted medications within the permitted range was considered to be persistent. Other secondary outcome measures included changes in: the Positive and Negative Syndrome Scale (PANSS)^9^; the Clinical Global Impression–Severity of illness (CGI‐S)^10^; the Global Assessment of Functioning (GAF)^11^; the Sheehan Disability Scale (SDS)^12^; and the Calgary Depression Scale for Schizophrenia (CDSS).^13^ Safety was assessed by occurrence of adverse events, vital signs, body weight, clinical laboratory tests including prolactin level, and Drug‐Induced Extrapyramidal Symptoms Scale (DIEPSS).^14^ Adverse events were classified using the Medical Dictionary for Regulatory Activities (MedDRA) v22.0 system organ class and preferred term.

### 2.4 Sample size

We expected that the medication persistence rate at 12 weeks in this study would be higher than that in the previous long‐term, single‐arm, open‐label study in Japan at 12 weeks.^7^ In the previous study, the discontinuation rate at 12 weeks was 35.6% in patients who switched from previously treated antipsychotics other than aripiprazole to brexpiprazole (results of post hoc calculation), so we set the predefined threshold for medication adherence at 12 weeks at 65%. Based on the medication persistence rates with atypical antipsychotics,^15^, ^16^ medication persistence rate at 12 weeks was set to the expected value of 80%. As the number of required patients was calculated to be 72 (80% power, 5% two‐sided significance level), target sample size was set to 75.

### 2.5 Target population

The full analysis set (FAS) comprised patients who were administered at least one dose of brexpiprazole, and efficacy data after the treatment were obtained. The safety analysis set comprised patients who were administered at least one dose of brexpiprazole, and at least one datum on safety after the treatment was obtained.

### 2.6 Statistical analyses

One sample *Z*‐test for a proportion was performed for persistence rates #1 and #2 at 12 weeks. The null hypothesis for these tests is that the population proportion of persistence rates is equal to a predefined threshold of 65%. Point estimates and 95% CIs were also calculated for persistence rates #1 and #2. Changes from baseline were tabulated for the following items: the SLOF, SWNS, PANSS (total, positive subscale, negative subscale, and general psychopathological subscale), CGI‐S, GAF, SDS, and CDSS. Summary statistics and 95% CIs were calculated for all these items, and *p*‐values (paired *t*‐test) for the SLOF and the SWNS.

Post hoc subgroup analyses of medication persistence rate #1 at 12 weeks were performed to calculate the point estimate and 95% CI for the following subgroup factors on the persistence rate: sex, age, body mass index (BMI), age at first onset, duration of illness, duration of antipsychotics, cohabitants, educational background, diagnosis, reason for switching, baseline PANSS total score, baseline CGI‐S, prior antipsychotics (olanzapine or risperidone/paliperidone), CP equivalent daily dose of prior antipsychotics, number of prior antipsychotics, and presence or absence of concomitant therapy.

We set the statistical significance level at 0.05. All tests were two‐tailed. All statistical analyses were conducted using SAS 9.4 32‐bit version for Windows (SAS Institute Inc., Cary, NC, USA).

### 2.7 Sensitivity analyses

Each sensitivity analysis was an analysis of the medication persistence rate, which was defined as non‐persistence if the following criteria were met in addition to the judgment of persistence or non‐persistence based on medication persistence rate #1. (1) Criteria in sensitivity analysis 1: met criteria for medication non‐persistence at 12 weeks with medication persistence rate #1, or worsened by at least 10 points from baseline by 12 weeks with a baseline PANSS total score of 40 or less, or worsened by at least 25% from baseline by 12 weeks with a baseline PANSS total score of 41 or more, or worsened by at least two steps from baseline on the CGI‐S. (2) Criteria in sensitivity analysis 2: met criteria for medication non‐persistence at 12 weeks with medication persistence rate #1, or any DIEPSS score (items 1–9) worsened from baseline to 4 at any time by 12 weeks, or DIEPSS score worsened by three or more steps from baseline. (3) Criteria in sensitivity analysis 3: met criteria for medication non‐persistence at 12 weeks with medication persistence rate #1 or met criteria for non‐persistence of medication in sensitivity analysis of 1 or 2.

## 3 RESULTS

### 3.1 Efficacy

**FIGURE 1:**
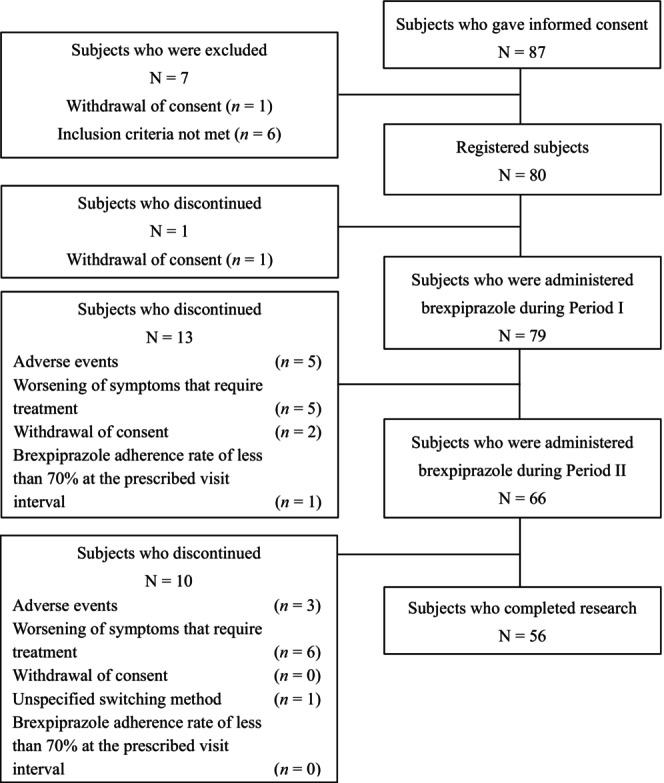
Patient disposition.

In this study, 87 patients were enrolled and screened for eligibility, and 79 were administered brexpiprazole. The FAS comprised 79 patients. During the period I, 13 (16.5%) patients discontinued brexpiprazole and 66 (83.5%) continued the drug. During period II, 10 (12.7%) patients discontinued treatment, and 56 (70.9%) completed the study (Figure 1). Major reasons for discontinuation were adverse events (*n* = 5, period I; *n* = 3, period II) and worsening of symptoms requiring treatment (*n* = 5, period I; *n* = 6, period II). Patient demographics and baseline characteristics are shown in Table 1. Patients were aged 47.9 ± 11.5 years (mean ± SD) and a minority were female (39.2%, *n* = 31). Body weight was 70.9 ± 13.7 kg (mean ± SD), and BMI was 26.1 ± 4.5 kg/m^2^ (mean ± SD). Regarding prior antipsychotics, 39 (49.4%) patients used olanzapine and 40 (50.6%) patients used risperidone/paliperidone. The major reasons for switching were tolerance issues (51 [64.6%] patients) and effectiveness issues (24 [30.4%] patients).

**TABLE 1:** Patient demographics and clinical characteristics at baseline.

Characteristic	*N* = 79
Age (years), mean ± SD	47.9 ± 11.5
Female sex, *n* (%)	31 (39.2)
Body weight (kg), mean ± SD	70.9 ± 13.7
BMI (kg/m^2^), mean ± SD	26.1 ± 4.5
BMI (kg/m^2^), *n* (%)	
<25	37 (46.8)
≥25	42 (53.2)
Educational background, *n* (%)	
Vocational school or university degree or higher	29 (36.7)
High school graduation	35 (44.3)
Junior high school graduation	15 (19.0)
Educated years (years), mean ± SD	12.7 ± 2.3
Diagnosis, *n* (%)	
Schizophrenia	77 (97.5)
Schizoaffective disorder	2 (2.5)
Age of onset (years), mean ± SD	31.1 ± 10.9
Duration of illness (years), mean ± SD	16.9 ± 12.3
PANSS total score, mean ± SD	57.8 ± 21.7
PANSS total score, *n* (%)	
< 60	45 (57.0)
60 ≤ and < 90	26 (32.9)
≥90	8 (10.1)
CGI‐S, mean ± SD	3.0 ± 1.1
CP equiv. of prior antipsychotics (mg/day), mean ± SD	469.4 ± 228.4
Prior antipsychotics (major agent), *n* (%)	
Olanzapine	39 (49.4)
Risperidone or Paliperidone	40 (50.6)
Reason for switching, *n* (%)	
Tolerance issues	51 (64.6)
Effectiveness issues	24 (30.4)
Others	4 (5.1)

Abbreviations: BMI, body mass index; CGI‐S, Clinical Global Impression–Severity of illness; CP, chlorpromazine; PANSS, Positive and Negative Syndrome Scale; SD, standard deviation.

Medication persistence rate #1 at Week 12 was 78.5% (95% CI: 67.8, 86.9; Table 2). The lower limit of the two‐sided 95% confidence interval was 67.8, which was higher than the predefined threshold of 65%. Sensitivity analyses showed similar trends with high medication persistence rates. The medication persistence rate #2 at Week 12 was exactly the same as medication persistence rate #1 because there was no case who used the concomitantly restricted medications within the permitted range. A significant improvement was observed in the mean change in the SLOF score from baseline in the subjects who completed the study at either Week 12 or 24 (Table 3); however, the last observation carried forward (LOCF) analysis, which included those who dropped out during the study, showed no significant improvement. A significant improvement was observed in the mean change in the SWNS score from baseline in subjects who completed the study at Week 12 but not at Week 24. Meanwhile, the LOCF analysis, which included those who dropped out during the study, showed no significant improvement. As for the other secondary outcome measures, a significant improvement was observed at either Week 12 or 24 in the subjects who completed the study for the PANSS total score as well as positive, negative, and general psychopathological subscale scores, CGI‐S, and GAF scores, whereas only at Week 24 was an improvement observed for the CDSS score (Table 4). Among these variables, the LOCF analysis, which included those who dropped out during the study, showed a significant improvement in the PANSS negative subscale score alone. In addition, there was no significant improvement at any timepoint in the SDS score. We also performed subgroup analysis of the medication persistence rate #1 at 12 weeks (Table S2). The results showed no substantial differences among subgroups for each factor analyzed.

**TABLE 2:** Medication persistence rates and their sensitivity analyses.

Evaluation items	No. of persistent patients/no. of cases to be analyzed	Medication persistence rate	95% CI (regular approximation)	Test of the population ratio with a threshold of 65% (regular approximation)
Medication persistence rate #1 (primary outcome measure)	62/79	78.5	[67.8, 86.9]	*p* < 0.0001
Sensitivity analysis 1 of medication persistence rate #1	62/79	78.5	[67.8, 86.9]	*p* < 0.0001
Sensitivity analysis 2 of medication persistence rate #1	61/79	77.2	[66.4, 85.9]	*p* < 0.0001
Sensitivity analysis 3 of medication persistence rate #1	61/79	77.2	[66.4, 85.9]	*p* < 0.0001
Medication persistence rate #2	62/79	78.5	[67.8, 86.9]	*p* < 0.0001

*Note*: Criteria for Medication persistence rate #1: Patients who were switched to brexpiprazole according to the specified switching method and had the brexpiprazole treatment period for at least 85 days were “medication persistence”; otherwise, “non‐persistence.” Sixty‐six patients completed Period I of the study, but four did not meet the switching method specified in the primary endpoint. As a result, the number of “medication persistence” was 62. Criteria for non‐persistence of medication in sensitivity analysis 1: Met criteria for medication non‐persistence at 12 weeks with medication persistence rate #1, or worsened by at least 10 points from baseline by 12 weeks with a baseline PANSS total score of 40 or less, or worsened by at least 25% from baseline by 12 weeks with a baseline PANSS total score of 41 or more, or worsened by at least two steps from baseline on the CGI‐S. Criteria for non‐persistence of medication in sensitivity analysis 2: Met criteria for medication non‐persistence at 12 weeks with medication persistence rate #1, or any DIEPSS score (items 1–9) worsened from baseline to 4 at any time by 12 weeks, or DIEPSS score worsened by three or more steps from baseline. Criteria for non‐persistence of medication in sensitivity analysis 3: Met criteria for medication non‐persistence at 12 weeks with medication persistence rate #1, or met criteria for non‐persistence of medication in sensitivity analysis of 1 or 2.

Abbreviations: CGI‐S, Clinical Global Impression–Severity of illness; CI, confidence interval; DIEPSS, Drug‐Induced Extrapyramidal Symptoms Scale; PANSS, Positive and Negative Syndrome Scale.

**TABLE 3:** Changes in SLOF and SWNS.

Evaluation items	*n*	Measured value					Change from BL						
Weeks		Mean	SD	Min	Med	Max	Mean	SD	Min	Med	Max	95% CI	Paired *t*‐test (*p*)
<SLOF>													
BL	79	89.6	16.3	50	90.0	120							
12	68	90.9	18.0	54	90.5	120	2.9	11.7	−38	3.0	35	[0.1, 5.7]	*p* = 0.0461
24	54	92.7	17.6	54	96.5	120	4.2	12.2	−32	2.0	45	[0.9, 7.5]	*p* = 0.0144
LOCF	75	89.4	19.6	32	91.0	120	0.3	15.1	−58	1.0	45	[−3.2, 3.8]	*p* = 0.8671
<SWNS>													
BL	79	74.1	17.1	33	72.0	118							
12	68	76.0	16.2	42	75.5	110	3.7	14.5	−29	3.0	38	[0.2, 7.2]	*p* = 0.0409
24	56	75.1	17.7	35	74.5	114	3.1	16.1	−35	2.0	49	[−1.2, 7.4]	*p* = 0.1560
LOCF	75	73.9	18.2	25	72.0	114	1.1	16.3	−51	1.0	49	[−2.6, 4.9]	*p* = 0.5533

Abbreviations: BL, baseline; CI, confidence interval; LOCF, last observation carried forward; Max, maximum; Med, median; Min, minimum; SD, standard deviation; SLOF, Specific Levels of Functioning Scale; SWNS, Subjective Well‐being under Neuroleptic drug treatment Short form.

**TABLE 4:** Changes in PANSS, CGI‐S, GAF, SDS, and CDSS scores.

Evaluation items	*n*	Measured value					Change from BL					
Weeks		Mean	SD	Min	Med	Max	Mean	SD	Min	Med	Max	95% CI
PANSS total score												
BL	79	57.8	21.7	30	56.0	122						
12	68	52.9	20.6	30	51.5	132	−5.2	8.3	−32	−3.0	11	[−7.2, −3.2]
24	54	48.2	16.9	30	46.0	91	−8.1	9.6	−31	−4.5	9	[−10.8, −5.5]
LOCF	77	55.9	24.5	30	51.0	132	−2.2	17.1	−31	−3.0	67	[−6.0, 1.7]
PANSS positive subscale score												
BL	79	12.2	5.5	7	11.0	30						
12	68	11.5	5.4	7	10.0	28	−0.8	2.3	−13	0.0	5	[−1.3, −0.2]
24	54	10.8	5.0	7	9.0	28	−1.1	3.4	−11	0.0	9	[−2.1, −0.2]
LOCF	77	12.9	7.1	7	10.0	37	0.6	5.4	−11	0.0	20	[−0.6, 1.9]
PANSS negative subscale score												
BL	79	16.6	7.3	7	16.0	37						
12	68	15.0	6.4	7	14.5	32	−1.7	2.8	−12	−1.0	5	[−2.3, −1.0]
24	54	13.6	5.9	7	13.5	32	−3.0	3.5	−12	−2.0	3	[−4.0, −2.1]
LOCF	77	15.1	7.3	7	14.0	37	−1.6	4.7	−12	−1.0	16	[−2.7, −0.5]
PANSS general psychopathological subscale score												
BL	79	29.0	11.1	16	27.0	70						
12	68	26.4	10.5	16	24.0	76	−2.8	4.5	−21	−1.5	6	[−3.9, −1.7]
24	54	23.8	7.6	16	21.0	42	−4.0	5.0	−20	−2.0	5	[−5.3, −2.6]
LOCF	77	27.9	12.2	16	26.0	76	−1.2	8.3	−20	−1.0	31	[−3.1, 0.7]
CGI‐S												
BL	79	3.0	1.1	1	3.0	5						
12	68	2.7	1.2	1	3.0	6	−0.3	0.7	−2	0.0	2	[−0.4, −0.1]
24	54	2.5	1.2	1	3.0	5	−0.4	0.7	−2	0.0	1	[−0.6, −0.2]
LOCF	77	2.9	1.4	1	3.0	7	−0.1	1.0	−2	0.0	4	[−0.3, 0.2]
GAF												
BL	79	60.6	16.2	29	58.0	92						
12	68	64.0	16.8	10	64.0	95	3.6	7.9	−31	2.0	35	[1.7, 5.5]
24	54	68.3	16.9	33	68.5	96	7.3	8.2	−5	5.0	35	[5.0, 9.5]
LOCF	77	62.6	19.8	10	62.0	96	1.8	13.9	−53	2.0	35	[−1.4, 4.9]
SDS												
BL	79	7.0	6.3	0	6.0	27						
12	68	6.7	6.6	0	4.5	24	−0.5	6.9	−15	0.0	15	[−2.2, 1.1]
24	56	6.9	6.9	0	6.0	23	0.4	6.9	−12	0.0	22	[−1.4, 2.3]
LOCF	75	8.2	7.9	0	6.0	30	0.8	7.6	−13	0.0	22	[−0.9, 2.6]
CDSS												
BL	79	2.3	2.6	0	1.0	10						
12	68	2.1	2.7	0	1.0	11	−0.3	2.5	−9	0.0	6	[−0.9, 0.3]
24	54	1.6	2.3	0	0.0	8	−0.8	2.4	−10	0.0	5	[−1.5, −0.1]
LOCF	77	2.2	3.2	0	1.0	17	−0.1	3.2	−10	0.0	13	[−0.8, 0.6]

Abbreviations: BL, baseline; CDSS, Calgary Depression Scale for Schizophrenia; CGI‐S, Clinical Global Impression–Severity of illness; CI, confidence interval; GAF, Global Assessment of Functioning; LOCF, last observation carried forward; Max, maximum; Med, median; Min, minimum; PANSS, Positive and Negative Syndrome Scale; SD, standard deviation; SDS, Sheehan Disability Scale.

### 3.2 Safety

Concerning safety, 64.6% of subjects experienced at least one adverse event (Table 5). Most adverse events were mild (41.8%), or moderate (17.7%), in severity. The incidence of serious adverse events was 5.1%, consisting of schizophrenia and hallucination. Adverse events leading to discontinuation were schizophrenia, insomnia, fatigue, and drug withdrawal syndrome. There were no deaths in the study. In terms of DIEPSS scores, there was little change, and the mean change from baseline in overall severity did not exceed 0.1 at any timepoint. The mean prolactin levels were high at baseline, 25.60 μg/L in males, and 73.48 μg/L in females and decreased significantly by 12 weeks after switching to brexpiprazole, to 9.43 μg/L in males and 20.51 μg/L in females and remained within the reference range thereafter (9.43 μg/L in males and 25.64 μg/L in females at 24 weeks). Weight loss of 7% or more from baseline was observed in 15.6% (*n* = 12) of patients, while weight gain was observed in 6.5% (*n* = 5) of patients. None of those who lost weight had a BMI less than 18.5 kg/m^2^, and only one (1/5) of those who gained weight had a BMI greater than 30 kg/m^2^.

**TABLE 5:** Safety indicators.

Safety analysis set	Entire test period (*N* = 79)	Period 1 (*N* = 79)	Period 2 (*N* = 66)
	*n* (%)	*n* (%)	*n* (%)
Incidence of TEAE, *n* (%)			
At least one TEAE	51 (64.6)	41 (51.9)	37 (56.1)
Any serious TEAE	4 (5.1)	2 (2.5)	2 (3.0)
Discontinuation due to TEAE	17 (21.5)	11 (13.9)	6 (9.1)
TEAE occurring in >5% of patients in any group, *n* (%)			
Nasopharyngitis	9 (11.4)	7 (8.9)	4 (6.1)
Akathisia	4 (5.1)	2 (2.5)	2 (3.0)
Insomnia	20 (25.3)	18 (22.8)	12 (18.2)
Schizophrenia	12 (15.2)	5 (6.3)	7 (10.6)

Abbreviation: TEAE, treatment‐emergent adverse event.

## 4 DISCUSSION

In this study, the medication persistence rate (78.5%) at 12 weeks was significantly higher than the predefined threshold (65%). A study that compared the discontinuation rates between three second‐generation antipsychotics, aripiprazole, blonanserin, and paliperidone, showed that the medication persistence rate at 12 weeks for each drug was 64.6%, 58.8%, and 64.3%, respectively.^17^ The study adopted a similar period for switching and dose adjustment, which was between 4 and 8 weeks. Even taking into account that 6% of the patients in the study^17^ were not receiving medication at baseline, the medication persistence rate after switching in our study was thought to be high enough. It was found that the success rate of switching was increased when the plateau cross‐titration method was used, with sufficient time and an appropriate dose.

Regarding the prior medication, the persistence rate at 12 weeks was 84.6% for olanzapine and 72.5% for risperidone/paliperidone, indicating that persistence rate was numerically higher in olanzapine than in risperidone/paliperidone. This was somewhat surprising, since it was generally assumed that switching from olanzapine, which has anticholinergic effects, was potentially more challenging due to the influence of cholinergic rebound.^5^, ^18^ It may be because the gradual reduction in the drug over a 6‐week period helped to reduce the rebound effect.^19^ The results of the sub‐analysis also showed that no other clinical and/or demographic variables showed a significant effect on the persistence rate for brexpiprazole after switching (Table S2). It was thought to be difficult to predict the outcome of the switch in advance.

The SLOF has been developed as an interview‐based multidimensional assessment measure of social functioning, including interpersonal, vocational, and independent‐living domains.^20^ Specifically, Harvey et al.^21^ found that the SLOF, among other social function scales, best‐predicted performance for either neurocognition or functional capacity. Interestingly, a significant improvement was observed in the subjects who completed the study for the SLOF but not for the SDS, which also reflects the level of social functioning. The discrepancy in results may be influenced by the difference that SLOF reflects functional capacity related to social functioning, while SDS preferentially reflects functional outcomes; SLOF items include those related to cognitive functioning in daily living, while SDS reflects the degree to which the disease has impaired social life. Even though functional capacity may improve with drug switching, it may take a little more time before improvements in functional outcomes become explicit.

The Subjective Well‐being under Neuroleptic (SWN) drug treatment^22^, ^23^ is one of the most widely used tools for assessing subjective well‐being—the major component of quality of life in patients with schizophrenia.^23^, ^24^, ^25^, ^26^ Among various antipsychotic‐related side effects, apathy and anhedonia, the so‐called neuroleptic‐induced deficit syndrome, have an important influence on the patient's subjective well‐being. Evidence suggests that the neuroleptic‐induced deficit syndrome might be caused by inhibition of the dopaminergic reward system. Because brexpiprazole is a dopamine D_2_ partial agonist, it does not strongly inhibit the dopaminergic reward system and is expected to improve the SWN. As such, significant improvement was observed in subjects who completed the study at 12 weeks but not at 24 weeks. It is also acknowledged that subjective well‐being is relative^27^, ^28^ and therefore sensitive to change. Presumably, the SWN improved due to the release of inhibition of the dopaminergic reward system immediately after the switch, but the initial positive responses did not last long after the change because habituation had occurred.

For other symptoms and functional assessments such as PANSS, CGI‐S, and GAF, those who completed the study showed significant improvement from baseline at 12 and 24 weeks after switching; for the symptom of depression in schizophrenia as assessed by the CDSS, the improvement was significant only at 24 weeks. This could be because the baseline value was low. Improvement was as expected given that the switch was successful, whereas significant improvement in PANSS negative symptom subscale scores was also observed in the LOCF analysis, including those who dropped out. This may be due to the effect of brexpiprazole as a dopamine D_2_ partial agonist and implies that brexpiprazole may improve motivation, which is essential for psychosocial therapy. It would be worthwhile to conduct further research on the effect of brexpiprazole on motivation, which may lead to the enhancement of treatment effects of psychosocial therapy.

The significant improvement in prolactin levels was presumably due to brexpiprazole's dopamine partial agonist effects. Because no significant weight changes were observed, and there were few cases of weight loss in lean subjects and weight gain in obese subjects, the metabolic side effects that are a concern with atypical antipsychotics^29^ were thought to be minimal in brexpiprazole. However, as a limitation of this study, this is not a blinded, comparative study and the enrolled patients may have had high expectations of switching medication to a new drug, which may have resulted in an inflated persistence rate and improvement in clinical indices. In addition, because patients taking more than 1000 mg/day of prior antipsychotic and aged >65 years old were not included, the results may not be generalizable to the entire population with schizophrenia.

## 5 CONCLUSIONS

Brexpiprazole was appropriately switched over a period of 8 weeks using a plateau cross‐titration method with a relatively long period for tapering off the prior antipsychotic drug. The persistence rate for patients whose prior antipsychotic was olanzapine was numerically higher than those for patients whose previous antipsychotic was risperidone/paliperidone, and we did not find any factors that showed substantial differences in the persistence rates among subgroups in each factor. Clinical indices such as social functioning capacity, subjective well‐being, and psychiatric symptoms showed significant improvement from baseline for those who were able to complete the study. Particularly, negative symptoms showed a significant improvement, including cases that dropped out of the study. There were no adverse events to be concerned about, and the prolactin levels showed significant improvements while body weight showed no significant effects. Brexpiprazole appeared to be an appropriate drug to switch to using a plateau cross‐titration method when the prior antipsychotic was deemed inappropriate.

## AUTHOR CONTRIBUTION

**Kazuyuki Nakagome:** Conceptualization; methodology; investigation; resources; data curation; writing—original draft; writing—review and editing; visualization; supervision. **Hisateru Tachimori:** Conceptualization; methodology; formal analysis; data curation; writing—original draft; writing—review and editing; visualization. **Shiro Endo and Ken Murakami:** Investigation; resources; data curation; writing—review and editing. **Takaharu Azekawa and Seiji Hongo:** Conceptualization; investigation; resources; data curation; writing—review and editing. **Kazunari Niidome and Sakiko Yamada:** Methodology; data curation; writing—original draft; writing—review and editing; visualization; funding acquisition. **Yoshitsugu Kojima:** Conceptualization; methodology; formal analysis; data curation; writing—original draft; writing—review and editing; visualization; funding acquisition. **Hideki Oi:** Conceptualization; methodology; data curation; writing—original draft; writing—review and editing; visualization; project administration. **Tomiki Sumiyoshi:** Conceptualization; methodology; data curation; writing—original draft; writing—review and editing.

## CONFLICT OF INTEREST STATEMENT

This study was funded by Otsuka Pharmaceutical Co., Ltd, Tokyo, Japan. KNa reports having received lecture fees from Otsuka Pharmaceutical Co., Ltd. and Sumitomo Pharma Co., Ltd. He has also received research funding from Otsuka Pharmaceutical Co, Ltd., Sumitomo Pharma Co., Ltd., and Janssen Pharmaceutical K.K. TS reports honoraria received for advisory boards/lectures/papers and/or research funding from Takeda Pharmaceutical Co., Ltd., Sumitomo Pharma Co., Ltd., Meiji Seika Pharma Co., Ltd., Otsuka Pharmaceutical Co., Ltd., Eli Lilly Japan K.K., Lundbeck Japan K.K., Ono Pharmaceutical Co., Ltd., Janssen Pharmaceutical K.K., Shionogi & Co., Ltd., and VeraSci Co., Ltd. HT belongs to an endowed course funded by Takeda Pharmaceutical Co., Ltd., and belongs to a department that accepts financial support from the National Clinical Database, Johnson & Johnson K.K., and Nipro Co. HO has received research funding from Otsuka Pharmaceutical Co., Ltd., and Sumitomo Pharma Co., Ltd. SE reports having received lecture fees from Janssen Pharmaceutical K.K., Meiji Seika Pharma Co., Ltd., Sumitomo Pharma Co., Ltd., and Otsuka Pharmaceutical Co., Ltd. KM reports having received lecture fees from Otsuka Pharmaceutical Co., Ltd., Eisai Co., Ltd., Sumitomo Pharma Co., Ltd., Eli Lilly Japan K.K., Takeda Pharmaceutical Co., Ltd., Shionogi & Co., Ltd., Meiji Seika Pharma Co., Ltd., and Lundbeck Japan K.K. TA reports having received lecture fees from Eli Lilly Japan K.K., Otsuka Pharmaceutical Co., Ltd., Eisai Co., Ltd., Sumitomo Pharma Co., Ltd., and Pfizer Japan Inc. SH reports having received lecture fee from Otsuka Pharmaceutical Co., Ltd. KNi, YK and SY are employees of Otsuka Pharmaceutical Co., Ltd. Tomiki Sumiyoshi and Kazuyuki Nakagome are Editorial Board members of Neuropsychopharmacology Reports and co‐ authors of this article. To minimize bias, they were excluded from all editorial decision‐making related to the acceptance of this article for publication.

## ETHICS STATMENT

Approval of the Research Protocol by an Institutional Reviewer Board: The protocol for this study has been approved by the ethics committee of the institution (National Center of Neurology and Psychiatry Clinical Research Review Board, ID# CRB3200004) within which the work was undertaken, and it conforms to the provisions of the Declaration of Helsinki.

Informed Consent: The patients who participated in this study gave informed consent and patient anonymity was preserved.

Registry and the Registration No. of the Study/Trial: This study is registered in Japan Registry of Clinical Trials (jRCTs031180015).

Animal Studies: N/A.

## Supporting information


Appendix S1.

## Data Availability

The data are not publicly available because the protocol approved by the ethics committee states that “any use of the data or data sets from this research will be conducted only after a new research protocol has been developed, reviewed, and approved by the Ethics Committee.”
