# Supplementary material for: A multicenter, single‐arm, open‐label interventional study of adherence to brexpiprazole during switching from previous antipsychotic drugs in patients with schizophrenia or schizoaffective disorder
Source: Neuropsychopharmacol Rep. 2024 Jan 22;44(1):187–96. doi: 10.1002/npr2.12416 (PMC10932803; doi:10.1002/npr2.12416)
Supplement: Supplementary file 1 — Appendix S1. [file NPR2-44-187-s001.docx]

Supplemental Figure S1. Switching procedure

10

6

8

4

2

0

12

Informed consent

24 weeks

1 mg

2 mg Brexpiprazole

Period I (switching and post-switch)

Period II (maintenance)

Prior antipsychotics

Supplemental Table S1. Exclusion criteria and withdrawal criteria

Exclusion criteria

(1) Patients who were pregnant or planning to become pregnant

(2) Patients with a history of clozapine use

(3) Patients with clinically significant neurological, hepatic (moderate to severe hepatic dysfunction (Child-Pugh classification B or C), renal, metabolic, hematological, immunological, cardiovascular, pulmonary, or gastrointestinal disorders. Medical conditions that were minor or well-controlled were considered acceptable if the condition did not interfere with assessments of safety or efficacy.

(4) Patients with HbA1c ≥ 6.5% on screening (NGSP: National Glycohemoglobin Standardization Program value) (JDS: Japan Diabetes Society value: 6.1%)

(5) Patients with the following laboratory values at screening

1. Platelet count ≤ 75000/mm^3^ (/μL)

2. Hemoglobin ≤ 9 g/dL

3. Neutrophils, absolute ≤ 1000/mm^3^

4. AST (aspartate aminotransferase) > 2 x Upper limit of normal (ULN)

5. ALT (alanine transaminase) > 2 x ULN

6. CPK (creatine phosphokinase) > 3 x ULN

7. Creatinine ≥ 2 mg/dL

(6) Patients who developed acute depressive symptoms within 30 days prior to obtaining informed consent and were judged to require treatment with antidepressants

(7) Patients who received electroconvulsive therapy within 60 days prior to obtaining consent

(8) Patients receiving treatment with a long-acting injectable antipsychotic within 90 days prior to obtaining consent

(9) Patients hospitalized for psychiatric symptoms within 90 days prior to obtaining consent

(10) Patients with suicidal ideation within 180 days prior to obtaining consent, suicidal behavior within the last two years, or were judged to be at a high risk of suicide

(11) Patients with substance abuse or dependence, including alcohol and benzodiazepines, within 180 days prior to obtaining consent

(12) Patients with contraindications to brexpiprazole

1. Patients in coma

2. Patients under the substantial influence of central nervous depressants, such as barbiturate derivatives and anesthetics

3. Patients receiving adrenaline

4. Patients with a history of hypersensitivity to brexpiprazole or any of its components

(13) Patients taking concomitant CYP2D6 inhibitors (quinidine, paroxetine, etc.) or strong CYP3A4 inhibitors (itraconazole, clarithromycin, etc.)

(14) Patients known to be deficient in CYP2D6 activity

(15) Patients who were judged by the principal investigator or the subinvestigator to be incapable of participating in this study safely

(16) Patients who had participated in a clinical trial of brexpiprazole in the past

(17) Patients who took brexpiprazole in the past

Withdrawal Criteria

(1) Developing an adverse event that requires discontinuation (including people in whom pregnancy was identified)

(2) Worsening of psychiatric symptoms requiring new antipsychotics (except provisions of concomitantly restricted medications use) or hospitalization

(3) Subject withdrew consent

(4) Lost to follow-up

(5) Violation of eligibility criteria was found after the initiation of the study.

(6) Failure to discontinue previously treated antipsychotic medication after 8 weeks of brexpiprazole administration

(7) Failure to increase the dose of brexpiprazole to 2 mg/day within 7days after the initiation of treatment

(8) Dose reduction to 1 mg/day after dose was increased to 2 mg/day of brexpiprazole

(9) Patient was adjudged by the principal investigator or subinvestigator to require discontinuation of the study due to an excessive mental or physical burden.

(10) Compliance with brexpiprazole was < 70% between the last scheduled visit and the current scheduled visit.

(11) Compliance with previously treated primary antipsychotic < 70% between the last scheduled visit and the current scheduled visit (until visit at 8 weeks)

(12) Patient was adjudged by the principal investigator or subinvestigator to require discontinuation of the study for any other reasons.

Supplemental Table S2. Subgroup analysis of medication persistence rate #1 at week 12

| FAS |  |  |  |  |
| --- | --- | --- | --- | --- |
|  |  | Patients who continued medication  /Patients analyzed |  | 95% CI |
| Evaluation items |  |  | Medication persistence rate | (Regular approximation) |
| Sex | Male | 38 / 48 | 79.2 | [65.0, 89.5] |
|  | Female | 24 / 31 | 77.4 | [58.9, 90.4] |
| Age (years) | < median (51.0) | 31 / 38 | 81.6 | [65.7, 92.3] |
|  | ≥ median | 31 / 41 | 75.6 | [59.7, 87.6] |
| BMI (kg/m^2^) | < median (25.56) | 33 / 40 | 82.5 | [67.2, 92.7] |
|  | ≥ median | 29 / 39 | 74.4 | [57.9, 87.0] |
| Age of onset (years) | < median (31.0) | 28 / 36 | 77.8 | [60.8, 89.9] |
|  | ≥ median | 34 / 43 | 79.1 | [64.0, 90.0] |
| Duration of illness (years) | < median (15.64) | 32 / 39 | 82.1 | [66.5, 92.5] |
|  | ≥ median | 30 / 40 | 75.0 | [58.8, 87.3] |
| Duration of antipsychotics | < median (15.14) | 32 / 40 | 80.0 | [64.4, 90.9] |
| （years by the time of informed consent） | ≥ median | 30 / 39 | 76.9 | [60.7, 88.9] |
| Cohabitants | Present | 47 / 62 | 75.8 | [63.3, 85.8] |
|  | Absent | 15 / 17 | 88.2 | [63.6, 98.5] |
| Educational background | Vocational school or university degree or higher | 25 / 29 | 86.2 | [68.3, 96.1] |
|  | High school graduation | 25 / 35 | 71.4 | [53.7, 85.4] |
|  | Junior high school graduation | 12 / 15 | 80.0 | [51.9, 95.7] |
| Diagnosis | Schizophrenia | 60 / 77 | 77.9 | [67.0, 86.6] |
|  | Schizoaffective disorder | 2 / 2 | 100.0 | [15.8, 100.0] |
| Reason for switching | Tolerance issue | 41 / 51 | 80.4 | [66.9, 90.2] |
|  | Effectiveness issue | 18 / 24 | 75.0 | [53.3, 90.2] |
|  | Others | 3 / 4 | 75.0 | [19.4, 99.4] |
| Baseline PANSS total score | < 60 | 37 / 45 | 82.2 | [67.9, 92.0] |
|  | 60 ≥ and < 90 | 19 / 26 | 73.1 | [52.2, 88.4] |
|  | ≥ 90 | 6 / 8 | 75.0 | [34.9, 96.8] |
| Baseline CGI-S | < 4 | 45 / 57 | 78.9 | [66.1, 88.6] |
|  | ≥ 4 | 17 / 22 | 77.3 | [54.6, 92.2] |
| Prior antipsychotics (major agent) | Olanzapine | 33 / 39 | 84.6 | [69.5, 94.1] |
|  | Risperidone or Paliperidone | 29 / 40 | 72.5 | [56.1, 85.4] |
| CP equiv. of prior antipsychotics (mg/day) | < 600 | 41 / 51 | 80.4 | [66.9, 90.2] |
|  | ≥ 600 | 21 / 28 | 75.0 | [55.1, 89.3] |
| Number of prior antipsychotics | One | 37 / 44 | 84.1 | [69.9, 93.4] |
|  | Two or more | 25 / 35 | 71.4 | [53.7, 85.4] |
| Concomitant therapy | Yes | 9 / 12 | 75.0 | [42.8, 94.5] |
|  | No | 53 / 67 | 79.1 | [67.4, 88.1] |
|  |  |  |  |  |

BMI, Body Mass Index; CGI-S, Clinical Global Impression–Severity of illness; CI, Confidence Interval; CP, chlorpromazine; FAS, Full Analysis Set; PANSS, Positive and Negative Syndrome Scale
